# Effects of the Stress of *Beauveria bassiana* on the Reproductive Success of an Idiobiont Parasitoid, *Sclerodermus guani*

**DOI:** 10.3390/insects17030278

**Published:** 2026-03-04

**Authors:** Yuenan Chen, Shasha Wu, Li Li, Hongmei Yao, Lilin Luo

**Affiliations:** 1 School of Life Sciences, Guizhou Normal University, Guiyang 550025, China; 2 Guizhou Key Laboratory of Forest Cultivation in Plateau Mountain, School of Life Science, Guizhou Normal University, Guiyang 550025, China; 3 Guizhou Institute of Biology, Guiyang 550009, China

**Keywords:** parasitic wasp, entomopathogen, *Monochamus alternatus*, invasion route, invasion timing

## Abstract

In the complex interplay among parasitic wasps, their insect hosts, and pathogenic microbes, the system involving *Sclerodermus guani* (Hymenoptera: Bethylidae) (a parasitoid wasp), *Monochamus alternatus* (Coleoptera: Cerambycidae) (the pine sawyer beetle, its host), and *Beauveria bassiana* (Hypocreales: Cordycipitaceae) (a fungus) presents a unique scenario where wasp offspring develop within a nearly sealed host gallery. This nursery is vulnerable to fungal invasion, often introduced by the foraging female wasps or *M. alternatus* itself, creating a three-way interaction where the fungus can infect both *M. alternatus* and *S. guani*. To assess how the route and timing of fungal exposure impact the *S. guani* population, we simulated this system by introducing different concentrations of *B. bassiana* either directly to the female wasps or to *M. alternatus* prior to parasitism. We further examined the effect of exposure timing by applying the fungus at different developmental stages of the *S. guani* offspring. Key population parameters, including the reproductive capacity of female wasps, the survival and developmental fitness of *S. guani* offspring and the germination period of hyphae, were measured. The results indicated that the most severe damage to populations of *S. guani* occurs when its host, *M. alternatus*, is infected by *B. bassiana*. Among the various developmental stages, *S. guani* offspring exhibited the greatest vulnerability during mid-to-late larval stages, whereas the egg and pupa within cocoon stages demonstrated a higher tolerance. We conclude that both the pathway and the timing of fungal exposure are critical factors influencing its impact. These findings provide valuable insights for optimizing the integrated use of biological agents in pest management, informing strategies that mitigate adverse effects on beneficial parasitoid wasps.

## Simple Summary

Within the complex interplay between an idiobiont ectoparasitoid, its beetle host, and a common fungus, we studied a scenario where *Sclerodermus guani* (Hymenoptera: Bethylidae) parasitizes and utilizes *Monochamus alternatus* (Coleoptera: Cerambycidae) within its sealed chamber. This nursery is often invaded by the *Beauveria bassiana* (Hypocreales: Cordycipitaceae), which hitchhikes on the female parasitoids or the host beetle. We investigated how different routes (via the female parasitoids of *S. guani* or the host beetle) and timing of *B. bassiana* exposure affect *S. guani* population dynamics and development. Our results showed that *B. bassiana* caused the most severe harm when it was carried by *M. alternatus*. The offspring of *S. guani* were also most vulnerable during their mid-to-late growth stages, while egg and pupa within cocoon stages were better protected. These differences are likely due to combined factors like maternal care, the offspring growth phase, and the nursery environment. The techniques parasitoids use to feed on and manipulate their hosts are ingenious, demonstrating multiple evolutionary pathways to achieve successful development from egg to adult. This study highlights that, in pest control, where *S. guani* and *B. bassiana* are used together, the pathway and timing of *B. bassiana* exposure are crucial to avoid harming the beneficial wasps, thereby improving the sustainable management of tree-boring *M. alternatus*.

## 1. Introduction

Pine wilt disease (PWD), caused by *Bursaphelenchus xylophilus* (Aphelenchida: Aphelenchoididae), severely threatens global pine forests. *Monochamus alternatus* Hope (Coleoptera: Cerambycidae) is the primary vector of *B. xylophilus* in Asia, and controlling this beetle is critical for limiting the spread of PWD [1]. Among biological control options, *Sclerodermus guani* Xiao et Wu (Hymenoptera: Bethylidae) serves as an important natural enemy of wood-boring pests, with the ability to penetrate bark and navigate frass-filled galleries to locate *M. alternatus* larvae and pupae [2]. Upon finding a suitable host, *S. guani* stings and paralytically immobilizes it, causing host death; the parasitoid then reproduces and rears its offspring on the host until they emerge [3,4,5]. Thus *S. guani* is particularly effective against this vector of pine wilt disease. However, its efficacy in biological control is shaped by multiple biotic and abiotic factors that influence reproduction and survival.

In natural ecosystems, organisms interact within complex networks [6,7]. *Sclerodermus guani* parasitizes its hosts inside their galleries, which form an almost sealed microenvironment and thus a confined arena for encounters between the parasitoid and the generalist insect pathogen *Beauveria bassiana* (Hypocreales: Cordycipitaceae) [8,9]. The introduction of the entomopathogenic fungus *B. bassiana* into this enclosed system, either by carrier wasps harboring conidia or infected host *M. alternatus* beetles, creates a multi-trophic interaction among *S. guani*, *M*. *alternatus*, and *B*. *bassiana*. In this context, *B. bassiana* acts as a natural enemy of *M. alternatus* while also posing a potential threat to *S. guani* [10,11].

Many studies show that *S. guani* does not passively endure pathogen threat. Instead, it has evolved tolerance or immune mechanisms that allow it to complete parasitization and reproduction even in environments containing certain concentrations of *B. bassiana* [11]. Female adults of *S. guani* carrying pathogens have also been investigated as a means to prevent *M. alternatus* [12]. However, the precise threshold of this tolerance remains unclear. The ecological effects of varying pathogen concentrations introduced by different pathways during distinct larval developmental stages on *S. guani* population reproduction are also unknown. Clarifying these points is essential to understand field population dynamics and to optimize *S. guani* efficacy in biological control. Therefore, under controlled experimental conditions, we simulated natural encounters between *S. guani* and *B. bassiana* across the parasitoid’s entire life cycle and systematically examined how *B*. *bassiana* invasion affects *S*. *guani* population reproduction. Pre-parasitizated *M. alternatus* beetles and adult female *S. guani* collected at different offspring developmental stages were exposed to three *B. bassiana* conidial suspension concentrations (1 × 10^4^, 1 × 10^5^ and 1 × 10^6^ conidia/mL) within this semi-enclosed parasitic microenvironment. We then systematically assessed maternal fecundity, progeny fitness, and progeny infection status.

## 2. Materials and Methods

### 2.1. Insects

The *S. guani* were raised by the Pest Control and Resource Utilization Laboratory of Guizhou Normal University (Guiyang, China) for 59 successive generations, and a stable experimental population was obtained. The larvae of *M. alternatus* were collected in Kaili City, Guizhou Province (107. 981 °E, 26. 566 °N); each *M. alternatus* individual was placed in a separate, ventilated 5 mL centrifuge tube (Nanjing Kaixin Biological Technology Co., Ltd., Nanjing, China) with sterile dried sawdust and refrigerated at 4–5 °C.

### 2.2. Beauveria bassiana Suspensions

The strain of *B. bassiana* (GZUIFR-AS1) was provided by the Institute of Fungus Resources, Guizhou University (Guiyang, China). *Beauveria bassiana* was seeded on PDA plates (d = 90 mm) (Nanjing Kaixin Biological Technology Co., Ltd., Nanjing, China) in the dark at 25 °C for 14 days [12]. The conidia were harvested with a sterile spatula, suspended in sterile distilled water supplemented with 0.05% Tween-80^®^ (Nanjing Kaixin Biological Technology Co., Ltd., Nanjing, China) aqueous solution, and mixed well on a vortex mixer (Nanjing Kaixin Biological Technology Co., Ltd., Nanjing, China). To count the conidia directly, take 10 μL of the suspension with a plate and repeat three times to get the mean. The final concentration of conidia was adjusted to 1 × 10^4^, 1 × 10^5^ and 1 × 10^6^ conidia/mL after dilution with sterile 0.05% Tween-80^®^ aqueous solution, and sterile water was used as the control. Germination in conidial suspensions was assessed prior to experiments and was always kept above 95% [13,14,15].

### 2.3. Infection of Monochamus alternatus with a Pathogen

Healthy *M. alternatus* larvae weighing 0.45–0.55 g were selected, first washed with clean water and then disinfected with 10% alcohol. Finally, they were washed with distilled water and soaked with filter paper to absorb excess water from the surface of the larvae [11,15,16]. They were then briefly immersed for 5 s in different concentrations of *B. bassiana* conidial suspensions (1 × 10^4^, 1 × 10^5^ and 1 × 10^6^ conidia/mL) to allow full coating of their bodies, after which they were air-dried again and placed in sterilized 35 mm diameter Petri dishes (Nanjing Kaixin Biological Technology Co., Ltd., Nanjing, China). A group treated with 0.05% Tween-80^®^ served as the control. Adult female wasps were then introduced into the culture dishes to host unit weight (1:0.1 g). The treated larvae were incubated at a constant temperature (25 ± 1 °C), controlled with humidity above 90% and 20 duplicates per concentration. Starting from the introduction of the wasps, observations were recorded every 24 h [13,14,15]. Biological parameters of the offspring survival under stress from *B. bassiana* conidial suspensions at different concentrations were determined and compared, including the reproductive capacity of female wasps, the average developmental period, survival rates, proportions, body weight of offspring, infection rate infection-related mortality rate of *S. guani* offspring, and germination period of hyphae [16,17].

The measurement methods for the relevant parameters are as follows:

The reproductive capacity of female wasps, eggs laid (eggs/clutch): the total number of offspring was defined as the count taken when no additional progeny of *S. guani* emerged from the host; and female progeny per female (females/female): the number of emerged female offspring/the total number of female wasps.

The average development period and the developmental stages of *S. guani* offspring are defined as follows: egg stage (ES), early-instar larva (EIL), late-instar larva (LIL), mature larva (ML), spinning mature larva (SML), and pupa within cocoon (PC) [17,18]. All durations are measured in days (d). Pre-oviposition period: the duration from the introduction of female wasps to the observation of the first egg on the host; egg duration: the developmental period from egg deposition to larval hatching; early instar larva stage: the developmental duration from the early instar to the late instar larva; late instar larva stage: the developmental duration from the late instar to the mature larva; mature larva stage: the duration from the mature larva to the spinning larva; spinning larva stage: the developmental period from the spinning larva to the pupal stage within the cocoon; pupal stage within the cocoon: the duration from pupation to the complete emerged of the offspring wasp; and whole generation: the total time from the deposition of the first egg by the female to the complete emerged of the offspring.

Egg survival rate (%) = (number of surviving eggs/total number of eggs laid) × 100, larval survival rate (%) = (number of surviving larvae/total number of eggs laid) × 100, pupal cocoon survival rate (%) = (number of emerged offspring/total number of eggs laid) × 100, and emergence rates (%) = (number of emerged offspring/number of pupal cocoons) × 100.

Proportions (%) = (number of male offspring/total number of emerged adult offspring) × 100, and body weight of offspring (mg) = the total weight of emerged female offspring/the number of emerged females.

Infection rate (%) = (number of plates with infected offspring/total number of plates inoculated) × 100, and infection-related mortality rate (%) = (number of plates with complete offspring mortality due to infection/total number of plates inoculated) × 100.

Hyphal germination period (d): the duration from the point of *B. bassiana* inoculation to the emergence of fungal hyphae [16,17,18].

### 2.4. Infection of Sclerodermus guani with a Pathogen

The developmental stages of *S. guani* offspring are defined as follows: egg stage (ES), early-instar larva (EIL), late-instar larva (LIL), mature larva (ML), spinning mature larva (SML), and pupa within cocoon (PC) [17].

Healthy *M. alternatus* larvae weighing 0.45–0.55 g were selected, surface-sterilized, air-dried, weighed, and transferred to clean 35 mm culture dishes as host units. Adult female wasps were introduced into the dishes to host unit weight (1:0.1 g).

Female wasps were subsequently and carefully removed at each of the six specified offspring developmental stages, and a separate group of naive female wasps (with no prior parasitic experience) creating seven experimental groups.

All female wasps were infected using the identical conidial suspension inoculation method described in Section 2.3 for infecting *M. alternatus* larvae.

The infected wasps removed at different offspring stages were returned to their original host-containing culture dishes. The infected naive wasps were transferred onto new, unparasitized fifth-instar *M. alternatus* larvae for standard rearing. Observations were recorded every 24 h. Biological parameters of the offspring survival under stress from *B. bassiana* conidial suspensions at different concentrations were determined and compared, including the reproductive capacity of female wasps, the average developmental period, survival rates, proportions, body weight, infection rate, infection-related mortality rate of *S. guani* offspring, and germination period of hyphae [16,17].

### 2.5. Data Analysis

Data were organized in Excel 2019 [19] and analyzed with IBM SPSS Statistics 27.0.1 [20]. The Kolmogorov–Smirnov test was used to determine whether the data follow a normal distribution. Analysis of variance (ANOVA) was applied, and comparisons of the mean values within each treatment group were conducted using the least significant difference (LSD) test. Figures were generated in Origin 2024 [21].

## 3. Results

### 3.1. Effects of Beauveria bassiana Stress on the Fecundity of the Adult Females Sclerodermus guani

**Figure 1:**
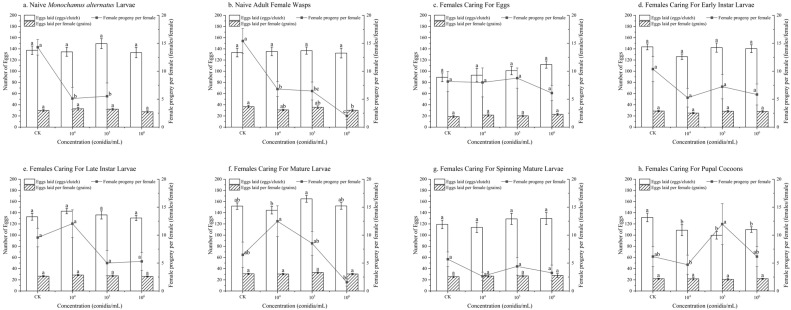
The reproductive capacity of female *Sclerodermus guani* under stress from *Beauveria bassiana* on *Monochamus alternatus* and different physiological states of *S. guani*. *Beauveria bassiana* infection stress on (**a**) naive *M. alternatus* larvae, (**b**) female wasps, (**c**) females caring for eggs, (**d**) females caring for early instar larvae, (**e**) females caring for late instar larvae, (**f**) females caring for mature larvae, (**g**) females caring for spinning mature larvae, and (**h**) females caring for pupal cocoons. Different letters above the bars indicate significant differences (mean ± SE, *n* = 20 in each treatment).

The control groups exhibited a higher number of female progeny per female compared to the experimental groups subjected to *B. bassiana* infection stress in the following treatments: stress on naive *M. alternatus* larvae (Figure 1a) and stress on naive adult female wasps (Figure 1b). Specifically, under *B. bassiana* infection stress on naive *M. alternatus* larvae, the female progeny per female at 1 × 10^4^ conidia/mL (5.12 ± 2.00 females/female) and 1 × 10^5^ conidia/mL (5.53 ± 2.37 females/female) was reduced by 9.17 and 8.76 females/female (*p* < 0.05), respectively, compared to the control (14.29 ± 1.41 females/female) (Figure 1a). No female offspring were produced under the high-concentration stress of 1 × 10^6^ conidia/mL (*p* < 0.05) (Figure 1a). Under *B. bassiana* infection stress on naive adult female wasps, the female progeny per female was 6.81 ± 1.33, 6.46 ± 1.61, and 2.01 ± 0.64 females/female at concentrations of 1 × 10^4^, 1 × 10^5^, and 1 × 10^6^ conidia/mL, respectively. These values were lower than that of the control group (15.41 ± 2.24 females/female), representing reductions of 8.60, 8.95, and 13.40 females/female, respectively (*p* < 0.01) (Figure 1b). Moreover, eggs laid per female was lower than the control (36.63 ± 2.19 grains) only at the treatment of 1 × 10^6^ conidia/mL (30.17 ± 1.94 grains) (*p* < 0.05) (Figure 1b). Notably, the female progeny per female remained below 3 females/female under the following conditions: at 1 × 10^6^ conidia/mL for both naive *M. alternatus* larvae and naive adult female wasps, at 1 × 10^6^ conidia/mL for females caring for mature larvae, and at 1 × 10^4^ conidia/mL for females caring for spinning mature larvae (*p* < 0.05) (Figure 1a,b,f). Furthermore, all experimental groups involving females caring for spinning mature larvae yielded fewer than 5 female progeny per female (*p* > 0.05) (Figure 1g).

The number of eggs laid under different treatments showed differences from the control group only in the experiments where *B. bassiana* infection stressed females during the pupal cocoon period. Specifically, the number of eggs laid at 1 × 10^4^ conidia/mL (108.80 ± 9.87 eggs/clutch), 1 × 10^5^ conidia/mL (99.67 ± 6.73 eggs/clutch) and 1 × 10^6^ conidia/mL (109.94 ± 4.94 eggs/clutch) was lower than that of the control group (131.64 ± 7.69 eggs/clutch) (*p* < 0.05) (Figure 1h).

### 3.2. Effects of Beauveria bassiana Stress on the Fitness of Sclerodermus guani Offspring

**Figure 2:**
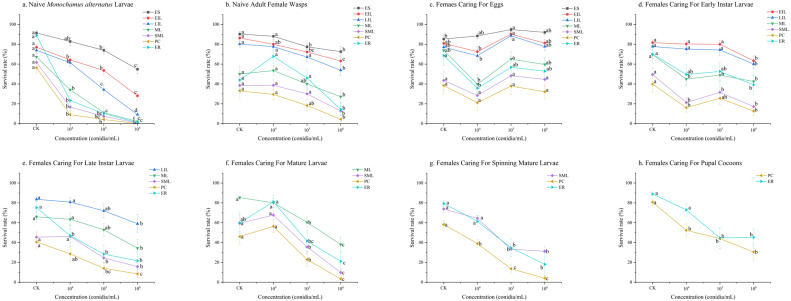
The survival rates of *Sclerodermus guani* offspring under stress from *Beauveria bassiana* on *Monochamus alternatus* and different physiological states of *S. guani*. *Beauveria bassiana* infection stress on (**a**) naive *M. alternatus* larvae; (**b**) female wasps; (**c**) females caring for eggs; (**d**) females caring for early instar larvae; (**e**) females caring for late instar larvae; (**f**) females caring for mature larvae; (**g**) females caring for spinning mature larvae; and (**h**) females caring for pupal cocoons. Different letters above the bars indicate significant differences (mean ± SE, *n* = 20 in each treatment). ES: eggs of *S. guani*, EIL: early instar larval, LIL: late instar larval, ML: mature larval, SML: spanning mature larval, PC: pupal cocoon, and ER: emergence rates [17].

The survival rates of *S. guani* offspring at various developmental stages under different treatments showed a general negative correlation with the concentration of *B. bassiana*; higher spore concentrations led to lower offspring survival rates. This trend was observed in all treatments except for those involving *B. bassiana* infection stress on females caring for eggs (Figure 2).

The most severe impact occurred following infection stress on naive *M. alternatus* larvae. At the highest concentration (1 × 10^6^ conidia/mL), the survival rates for late instar larvae (LIL = 9.19 ± 4.04%), mature larvae (ML = 2.24 ± 1.83%), spinning mature larvae (SML = 0.66 ± 0.48%), pupae within cocoons (PC = 0.05 ± 0.05%), and emergence rates (ER = 1.06 ± 1.06%) all fell below 10% (*p* < 0.01), representing reductions of 64.70%, 64.91%, 61.04%, 56.31% and 87.74%, respectively (Figure 2a). A secondary level of negative impact was observed when introduction occurred via naive adult female wasps, females caring for late instar larvae, mature larvae and spinning mature larvae. Specifically, at a concentration of 1 × 10^6^ conidia/mL, for the infection of naive adult female wasps, the survival rates were: SML = 12.79 ± 4.27%; PC = 4.46 ± 2.16%; ER = 14.34 ± 6.27% (*p* < 0.01) (Figure 2b). For the infection of females caring for late instar larvae, the survival rates were: SML = 15.85 ± 5.51%; PC = 8.42 ± 3.91%; ER = 21.67 ± 8.86% (*p* < 0.05) (Figure 2e). For the infection of females caring for mature larvae, the survival rates were: SML = 9.89 ± 3.42%; PC = 3.37 ± 1.62%; ER = 21.10 ± 8.70% (*p* < 0.01) (Figure 2g). For the infection of females caring for spinning mature larvae, the survival rates were: SML = 31.24 ± 5.11%; PC = 4.07 ± 2.34%; ER = 18.09 ± 11.52% (*p* < 0.01) (Figure 2g). In contrast, the introduction of the pathogen via females caring for eggs (*p* > 0.05) (Figure 2c) and pupal cocoons (*p* < 0.01) (Figure 2h) had the least negative effect on the *S. guani* population, with pupal cocoon survival rates all above 30% and emergence rates all above 45%.

**Figure 3:**
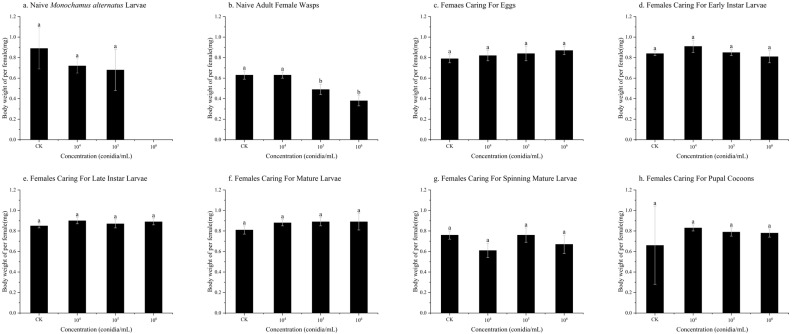
The body weight of *Sclerodermus guani* offspring under stress from *Beauveria bassiana* on *Monochamus alternatus* and different physiological states of *S. guani*. *Beauveria bassiana* infection stress on (**a**) naive *M. alternatus* larvae, (**b**) female wasps, (**c**) females caring for eggs, (**d**) females caring for early instar larvae, (**e**) females caring for late instar larvae, (**f**) females caring for mature larvae, (**g**) females caring for spinning mature larvae, and (**h**) females caring for pupal cocoons. Different letters above the bars indicate significant differences (mean ± SE, *n* = 20 in each treatment).

Among the different treatments, the difference in body weight per female was observed only in the group subjected to *B. bassiana* infection stress on naive adult female wasps. In this treatment, the body weight per female at 1 × 10^5^ conidia/mL (0.49 ± 0.05 mg) and 1 × 10^6^ conidia/mL (0.38 ± 0.05 mg) decreased by 0.14 mg and 0.25 mg, respectively, compared to the control group (0.63 ± 0.04 mg) (*p* < 0.01) (Figure 3b).

**Figure 4:**
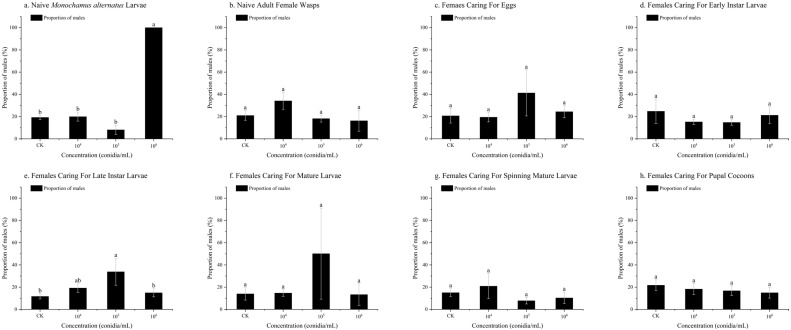
The proportions of *Sclerodermus guani* offspring under stress from *Beauveria bassiana* on *Monochamus alternatus* and different physiological states of *S. guani*. *Beauveria bassiana* infection stress on (**a**) naive *M. alternatus* larvae, (**b**) female wasps, (**c**) females caring for eggs, (**d**) females caring for early instar larvae, (**e**) females caring for late instar larvae, (**f**) females caring for mature larvae, (**g**) females caring for spinning mature larvae, and (**h**) females caring for pupal cocoons. Different letters above the bars indicate significant differences (mean ± SE, *n* = 20 in each treatment).

Among the different treatments, a difference in the proportion of males was observed only in the group subjected to *B. bassiana* infection stress on naive *M. alternatus* larvae and females caring for late instar larvae. Specifically, no female progeny was produced when naive *M. alternatus* larvae were stressed with *B. bassiana* at 1 × 10^6^ conidia/mL (*p* < 0.01) (Figure 4a). When females caring for late instar larvae were stressed with *B. bassiana* at 1 × 10^5^ conidia/mL, the proportion of male offspring (33.84 ± 12.08%) was higher by 22.13% than that in the control group (11.71 ± 2.26%) (*p* < 0.05) (Figure 4e).

Stress treatments targeting maternal wasps at different brood-care stages induced a biphasic shift in offspring development characterized by “early acceleration and later delay”. The larval stage, especially the early phase, was generally accelerated. In most treatments, the durations of both the early and late instar larval periods were shortened. For example, after *B. bassiana* infection stress on naive adult female wasps, the early instar larval periods under 1 × 10^4^ conidia/mL (1.10 ± 0.16 d), 1 × 10^5^ conidia/mL (0.90 ± 0.10 d), and 1 × 10^6^ conidia/mL (1.05 ± 0.05 d) were shortened by 0.85 d, 1.05 d, and 0.9 d, respectively, compared to the control (1.95 ± 0.20 d). The late instar larval period under 1 × 10^6^ conidia/mL (2.11 ± 0.14 d) was shortened by 0.79 d compared to the control (2.90 ± 0.14 d) (*p* < 0.01). After *B. bassiana* infection stress on females caring for eggs, the late instar larval period under 1 × 10^6^ conidia/mL (1.63 ± 0.17 d) was shortened by 0.84 d compared to the control (2.47 ± 0.27 d) (*p* < 0.01). Following *B. bassiana* infection stress on females caring for early instar larvae, the late instar larval periods under 1 × 10^4^ conidia/mL (1.47 ± 0.12 d) and 1 × 10^5^ conidia/mL (1.65 ± 0.18 d) were shortened by 0.74 d and 0.56 d, respectively, compared to the control (2.21 ± 0.12 d) (*p* < 0.01) (Table 1).

The mature larval period, which serves as the final transitional stage from larva to pupa, showed an inconsistent response but was predominantly prolonged. After *B. bassiana* infection stress on females caring for eggs, the mature larval periods under 1 × 10^4^ conidia/mL (4.46 ± 0.37 d), 1 × 10^5^ conidia/mL (4.31 ± 0.22 d), and 1 × 10^6^ conidia/mL (5.24 ± 0.33 d) were prolonged by 1.53 d, 1.38 d, and 2.31 d, respectively, compared to the control (2.93 ± 0.42 d) (*p* < 0.01). After *B. bassiana* infection stress on females caring for early instar larvae, the mature larval periods under 1 × 10^4^ conidia/mL (4.60 ± 0.19 d), 1 × 10^5^ conidia/mL (4.20 ± 0.24 d), and 1 × 10^6^ conidia/mL (4.38 ± 0.22 d) were prolonged by 2.13 d, 1.73 d, and 1.91 d, respectively, compared to the control (2.47 ± 0.17 d) (*p* < 0.01) (Table 1).

Across all treatments, the pupal cocoon period of the offspring showed prolongation. The most substantial extension, reaching 9.51 d, occurred under *B. bassiana* infection stress on naive adult female wasps at 1 × 10^6^ conidia/mL (treatment group = 31.36 ± 1.27 d vs. control = 21.85 ± 0.70 d) (*p* < 0.01). Although the early larval developmental stages were shortened, the prolongation of the mature larval period and especially the pupal cocoon period played a dominant role. Consequently, the whole generation (WG) development duration was longer in all treatment groups compared to their respective controls. Specifically, under stress on females caring for eggs and on naive adult female wasps at 1 × 10^6^ conidia/mL, the total development duration was extended by 4.58 d and 5.71 d, respectively (*p* < 0.01) (Table 1).

### 3.3. Effects of Beauveria bassiana Stress on the Infection Status of Sclerodermus guani Offspring

**Figure 5:**
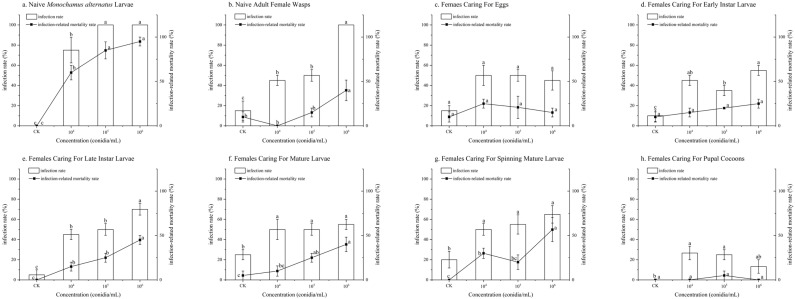
The infection rate and infection-related mortality rate of *Sclerodermus guani* offspring under stress from *Beauveria bassiana* on *Monochamus alternatus* and different physiological states of *S. guani*. *Beauveria bassiana* infection stress on (**a**) naive *M. alternatus* larvae, (**b**) female wasps; (**c**) females caring for eggs, (**d**) females caring for early instar larvae, (**e**) females caring for late instar larvae, (**f**) females caring for mature larvae, (**g**) females caring for spinning mature larvae, and (**h**) females caring for pupal cocoons. Different letters above the bars indicate significant differences (mean ± SE, *n* = 20 in each treatment).

Under stress from *B*. *bassiana* on naive *M*. *alternatus* larvae, naive female wasps, and females caring for late instar larvae, mature larvae, and spinning mature larvae, the infection rate and infection-related mortality rate of *S*. *guani* offspring showed a significant dose-dependent increase with rising concentrations of *B*. *bassiana*. The most severe effects were observed following *B*. *bassiana* stress on naive *M*. *alternatus* larvae: at a concentration of 1 × 10^4^ conidia/mL, the infection rate was 75.00 ± 12.58%, and the infection-related mortality rate was 60.00 ± 8.17%; at concentrations of 1 × 10^5^ conidia/mL and 1 × 10^6^ conidia/mL, the infection rates both reached 100.00 ± 0.00%, and the infection-related mortality rates increased to 85.00 ± 9.57% and 95.00 ± 5.00%, respectively (*p* < 0.01) (Figure 5a). Under *B*. *bassiana* stress on naive female wasps, although the infection rates reached 45.00 ± 5.00% and 100.00 ± 0.00% at low (1 × 10^4^ conidia/mL) and high (1 × 10^6^ conidia/mL) concentrations, respectively, the infection-related mortality rates were only 0.00 ± 0.00% and 40.00 ± 11.55% (*p* < 0.01) (Figure 5b). Under *B*. *bassiana* stress on females caring for spinning mature larvae, the infection-related mortality rate at the high concentration (1 × 10^6^ conidia/mL) reached the highest value among all caring groups, with an infection-related mortality rate of 56.67 ± 13.47% despite an infection rate of only 65.00 ± 8.66% (*p* < 0.05) (Figure 5g). These findings are also consistent with the results shown in Figure 2.

Under *B*. *bassiana* stress on females caring for early instar larvae and pupal cocoons, the infection rate of *S*. *guani* offspring showed a significant dose-dependent increase; however, the infection-related mortality rate did not increase with rising concentrations of *B*. *bassiana*. Under *B*. *bassiana* stress on females caring for early instar larvae, the infection rates at concentrations of 1 × 10^5^ conidia/mL and 1 × 10^6^ conidia/mL were 35.00 ± 5.00% and 55.00 ± 5.00%, respectively (*p* < 0.01), with infection-related mortality rates both below 30.00% (*p* > 0.05) (Figure 5d). Under *B*. *bassiana* stress on females caring for pupal cocoons at different concentrations, the infection rates were all below 30%, and the infection-related mortality rates were all below 6.00% (*p* > 0.05) (Figure 5h).

**Figure 6:**
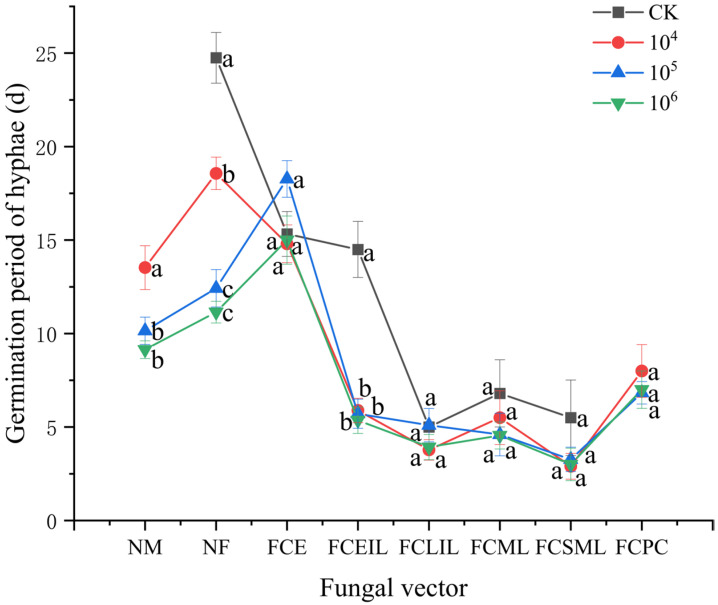
The germination period of hyphae of *Beauveria bassiana* following its introduction into the *Sclerodermus guani* population via different fungal vectors. NF: naive adult female wasps, NM: naive *Monochamus alternatus* larvae, FCE: females caring for eggs, FCEIL: females caring for early instar larvae, FCLIL: females caring for late instar larvae, FCML: females caring for mature larvae, FCSML: females caring for spinning mature larvae, and FCPC: females caring for pupal cocoons [22]. Different letters above the bars indicate significant differences (mean ± SE, *n* = 20 in each treatment).

Differences exist in the germination period of hyphae among different vectors and different physiological states of *S. guani* female wasps. Specifically, the hyphal germination period exceeded 9 d for carriers NM (*p* < 0.01), NF (*p* < 0.01), and FCE (*p* < 0.05). However, under different concentrations of *B. bassiana* stress, it was within 6 d for carriers FCEIL (*p* < 0.01), FCLIL (*p* > 0.05), FCML (*p* > 0.05), and FCSML (*p* > 0.05), and it was within 9 d for carrier FCPC (*p* < 0.01). For example, at 1 × 10^4^ conidia/mL, the germination period for *B. bassiana* carried by naive adult female wasps (NF) was 18.57 ± 0.87 d, which was 15.67 d longer than that for wasps caring for spinning mature larvae (FCSML) (2.90 ± 0.69 d). At 1 × 10^5^ conidia/mL, the germination period for wasps caring for eggs (FCE) was 18.27 ± 0.98 d, which was 15.00 d longer than that for wasps caring for spinning mature larvae (FCSML) (3.27 ± 0.65 d) (Figure 6).

Under stress from *B*. *bassiana* on naive *M*. *alternatus* larvae (NM), naive female wasps (NF), and females caring for early instar larvae, the effect of spore concentration on the germination period follows a pattern where the germination period gradually shortens as the spore concentration increases. After *B*. *bassiana* infection stress on NM at 1 × 10^4^, 1 × 10^5^, and 1 × 10^6^ conidia/mL, the germination periods were 13.53 ± 1.17 d, 10.15 ± 0.73 d, and 9.14 ± 0.47 d, respectively (*p* < 0.01). After stress from *B. bassiana* at concentrations of 1 × 10^4^, 1 × 10^5^, and 1 × 10^6^ conidia/mL on naive female wasps, the germination periods of hyphae were 18.57 ± 0.87 d, 12.42 ± 1.00 d, and 11.15 ± 0.58 d, respectively, compared to 24.75 ± 2.36 d in the control group (*p* < 0.01). After stress from *B. bassiana* at concentrations of 1 × 10^4^, 1 × 10^5^, and 1 × 10^6^ conidia/mL on females caring for early instar larvae, the germination periods of hyphae were 5.89 ± 0.65, 5.71 ± 0.78, and 5.36 ± 0.70 d, respectively, compared to 14.50 ± 1.50 d in the control group (*p* < 0.01) (Figure 6).

## 4. Discussion

In pest biological control, the simultaneous application of multiple natural enemies has emerged as a crucial strategy to improve control efficacy [23]. However, the interactions among different biological control agents are complex and variable, potentially resulting in synergistic, antagonistic, or neutral effects [24]. This study focuses on the typical interaction system of *S. guani*, *M. alternatus*, and *B. bassiana*, confirming the asymmetric influence of infection routes on *S. guani* population.

The population decline caused by *M. alternatus* infection was significantly greater than that from female wasp infection, a difference explained by resource competition theory [25]. When *B. bassiana* enters the system through the host, the host serves not only as a nutrient substrate for the pathogen but also as the exclusive resource pool for the development of parasitoid offspring. Rapid fungal proliferation deteriorates host quality, and fungal metabolites may directly harm parasitoid larvae. Guo et al. [10] found that *B. bassiana* pathogenicity increases with host size, linking host resources to fungal growth. At 1 × 10^6^ conidia/mL, the *S. guani* population nearly collapsed—no female offspring emerged, and the emergence rate was only 1.06 ± 1.06%. This highlights the potential severity of antagonism among biological control agents. Mechanistically, high-density conidia on the host surface allow *B. bassiana* to establish infection before parasitoid oviposition. Guo et al. [10] reported a lethal time of 7–14 days for *M. alternatus*, while *S. guani* larvae hatch within 4–5 days post-oviposition. By the time parasitoid larvae begin feeding, the host is already heavily infected. In contrast, conidia introduced via female wasps are limited, and *S. guani* can defend against infection through cleaning behaviors [11,26] and immune responses [11]. This explains the higher survival rates observed in the female wasp infection group. From an evolutionary perspective, this asymmetry reflects a fundamental conflict between parasitoids and pathogens over shared host resources [27,28].

Another key finding of this study is the differential sensitivity of offspring at various developmental stages to stress from *B. bassiana*. The egg and pupal cocoon stages were relatively tolerant, whereas late instar larvae, mature larvae, and spinning mature larvae exhibited high sensitivity. This pattern, which aligns with life history trade-off theory [29], suggests that organisms facing resource limitations must allocate resources among competing functional traits. Shi and Feng [30] assessed the infectivity of *B. bassiana*, *Metarhizium anisopliae* (Metschn.) Sorokin (Hypocreales: Clavicipitaceae), and *Paecilomyces fumosoroseus* (Wize) Kepler et al. (Hypocreales: Cordycipitaceae) against eggs of the spider mite *Tetranychus cinnabarinus* (Boisduval, 1867) (Trombidiformes: Tetranychidae), revealing that conidial infection rates on mite eggs were low. Li et al. [31] reported that the infection rate of *Metarhizium flavoviride* W. Gams & Rozsypal, 1973 (Hypocreales: Clavicipitaceae) against *Nilaparvata lugens* (Stål, 1854) (Hemiptera: Delphacidae) eggs was significantly lower than against adults. Several protective mechanisms may explain this: the chorion acts as a physical barrier obstructing conidial penetration, while low metabolic activity during the embryonic stage diminishes the active interfaces necessary for fungal infection [32]. When detecting pathogenic stress, females may transfer immune-related factors (e.g., antimicrobial peptides, vitellogenin) to offspring through the yolk, endowing them with pre-adaptive capabilities [33]. Research by Huang and Li [4] confirmed that maternal care significantly enhances offspring developmental performance, indicating that maternal protective behaviors during the egg and early larval stages are crucial for offspring survival. Li et al. [31] utilized *M. flavoviride* to infect different developmental stages of *N. lugens* and found that the order of virulence was adults > nymphs. The nymphal stage is protected by the cuticle and requires multiple molts, which may allow them to shed *M. flavoviride* conidia and thus reduce population mortality. Our study indicates that the high tolerance during the pupal cocoon stage may also stem from the physical isolation provided by the cocoon and the highly sclerotized pupal cuticle [34].

Portilla et al. [35] investigated the infection of different developmental stages of *Nezara viridula* (Linnaeus, 1758) (Hemiptera: Pentatomidae) with *B. bassiana* and found that mid-to-late fourth instar nymphs were the most susceptible, followed by second, third, and fifth instar nymphs. This is consistent with our findings. Herzner et al. [36] discovered that parental investment in antimicrobial defense is a crucial component of parental care, and this investment entails significant costs. The late instar larval stage of *S. guani* coincides with a critical period for rapid growth and energy reserve accumulation [32], during which resources are preferentially allocated to growth rather than immune defense, leading to increased susceptibility to pathogenic stress. Following the late instar larval stage, maternal care behaviors shift from grooming and nest cleaning activities to group transfer [5,18,26,37], resulting in a relative reduction in protective intensity, which closely aligns with the timing of peak susceptibility. Concurrently, the accumulation of larval feces and enhanced metabolic activity increase humidity within the nest, creating a microenvironment conducive to the growth of *B. bassiana* [38]. Consequently, after females caring for late instar, mature, and spinning mature larvae were subjected to *B. bassiana* stress, the germination period of hyphae was less than 6 d in all cases.

In most treatment groups, early and late instar larval durations were shortened, consistent with Wei et al. [11]. This may reduce exposure time to high-risk environments, though at the cost of compromised developmental quality (reduced body weight and survival). However, pupal and total developmental duration were prolonged under *B. bassiana* stress, differing from Wei et al. [14]. Hassan et al. [39]’s research indicated that the foraging activity of *Reticulitermes chinensis* Snyder, 1923 (Blattodea: Rhinotermitidae) termites infected with *B. bassiana* was significantly hindered; similarly, infected *S. guani* larvae may experience reduced metabolic activity and feeding, slowing development. Extending the pupal stage may provide additional time for tissue remodeling and energy reserve accumulation under nutritional or physiological stress. Wei et al. [14] reported adaptive reproductive strategies of *S. guani* under *B. bassiana* stress, including oviposition adjustments and offspring reallocation. The developmental duration changes observed here may represent a continuation of this adaptive strategy: after perceiving pathogenic stress, female wasps may regulate offspring developmental rhythm to maximize reproductive success.

In conclusion, from the perspective of integrated pest management (IPM) [23], this study demonstrates that assessing the potential risks of entomopathogenic fungi to natural enemy insects requires moving beyond simplistic “binary” toxicity tests. It is essential to consider two key contextual factors: the natural infection pathway and the developmental timing of the natural enemy. To avoid antagonistic effects when using *S. guani* as a carrier of *B. bassiana* for the combined control of *M. alternatus*, a spatiotemporally staggered strategy is recommended. Ultimately, successful multi-species biological control strategies depend on a comprehensive understanding and precise regulation of the intricate, dynamic interaction networks among the biological agents within the target ecological niche. This study provides important empirical evidence and a theoretical framework for this endeavor.

The differential effects observed in this study, stemming from various stress pathways and timings, may arise from intricate maternal influences and social regulation at the population level [40,41]. Direct stress on maternal wasps could elicit a heightened transgenerational resistance. When maternal wasps detect pathogen stress, they may transfer immune-related factors, such as antimicrobial peptides and vitellogenin along with antioxidants, or specific hormones, to their offspring through the yolk [11,42]. This transfer may confer “immune priming” or pre-adaptation, potentially resulting in increased survival rates during subsequent encounters with lower doses or later stages of pathogen exposure. Conversely, the health of maternal wasps and their brood care behaviors are essential for sustaining microbial homeostasis within the nest, representing a form of “social immunity” [17]. Therefore, future studies should explore the immune mechanisms of *S*. *guani* through research in molecular ecology and behavioral ecology.

## Figures and Tables

**Table 1:** The average developmental period of *Sclerodermus guani* under stress from *Beauveria bassiana* on *Monochamus alternatus* and different physiological states of *S. guani*.

Treatment	Concentration (Conidia/mL)	POP	Mean Developmental Duration of Offspring (d)						
			ES	EIL	LIL	ML	SML	PC	WG
NM	CK	4.30 ± 0.15 ab	4.85 ± 0.08 a	1.50 ± 0.11 a	3.00 ± 0.23 a	3.10 ± 0.20 a	1.00 ± 0.15 a	17.75 ± 0.24 c	30.60 ± 0.43 bc
	1 × 10^4^	4.43 ± 0.13 a	4.52 ± 0.11 a	1.75 ± 0.12 a	3.45 ± 0.23 a	3.60 ± 0.34 a	1.60 ± 0.35 a	19.22 ± 0.28 b	32.67 ± 0.41 ab
	1 × 10^5^	4.10 ± 0.10 b	4.55 ± 0.14 a	1.55 ± 0.11 a	3.56 ± 0.33 a	2.71 ± 0.47 a	1.40 ± 0.40 a	20.00 ± 1.00 ab	29.67 ± 0.88 c
	1 × 10^6^	4.00 ± 0.00 b	4.80 ± 0.12 a	1.63 ± 0.14 a	3.56 ± 0.24 a	2.50 ± 0.50 a	1.00 ± 1.00 a	21.00 ± 0.00 a	31.00 ± 0.89 a
NF	CK	4.20 ± 0.09 a	4.25 ± 0.12 a	1.95 ± 0.20 a	2.90 ± 0.14 a	3.06 ± 0.31 a	1.31 ± 0.24 a	21.85 ± 0.70 c	35.38 ± 0.50 bc
	1 × 10^4^	4.15 ± 0.08 a	3.95 ± 0.05 b	1.10 ± 0.16 b	2.50 ± 0.21 ab	2.20 ± 0.31 ab	0.95 ± 0.14 a	22.78 ± 0.46 c	33.39 ± 0.58 c
	1 × 10^5^	4.25 ± 0.10 a	4.00 ± 0.10 ab	0.90 ± 0.10 b	2.65 ± 0.15 a	1.71 ± 0.25 b	0.93 ± 0.32 a	26.87 ± 1.43 b	37.00 ± 1.12 b
	1 × 10^6^	4.05 ± 0.05 a	4.10 ± 0.10 ab	1.05 ± 0.05 b	2.11 ± 0.14 b	2.23 ± 0.32 ab	0.73 ± 0.14 a	31.36 ± 1.27 a	41.09 ± 1.19 a
FCE	CK	4.44 ± 0.20 a	4.22 ± 0.15 b	2.24 ± 0.24 a	2.47 ± 0.27 a	2.93 ± 0.42 b	2.15 ± 0.48 a	19.08 ± 0.42 b	32.42 ± 0.57 b
	1 × 10^4^	4.24 ± 0.14 a	5.12 ± 0.15 a	2.47 ± 0.17 a	2.35 ± 0.19 a	4.46 ± 0.37 a	2.18 ± 0.42 a	21.38 ± 0.71 a	37.00 ± 0.91 a
	1 × 10^5^	4.06 ± 0.06 a	5.28 ± 0.11 a	2.18 ± 0.21 a	2.71 ± 0.17 a	4.31 ± 0.22 a	1.93 ± 0.30 a	20.08 ± 0.46 ab	36.23 ± 0.38 a
	1 × 10^6^	4.32 ± 0.13 a	5.11 ± 0.13 a	2.63 ± 0.16 a	1.63 ± 0.17 b	5.24 ± 0.33 a	1.76 ± 0.48 a	20.94 ± 0.53 a	37.00 ± 0.48 a
FCEIL	CK	-	-	1.89 ± 0.17 a	2.21 ± 0.12 a	2.47 ± 0.17 b	1.35 ± 0.32 a	18.35 ± 0.60 b	31.59 ± 0.51 b
	1 × 10^4^	-	-	2.16 ± 0.12 a	1.47 ± 0.12 c	4.60 ± 0.19 a	1.21 ± 0.19 a	20.00 ± 0.36 a	34.79 ± 0.43 a
	1 × 10^5^	-	-	2.05 ± 0.14 a	1.65 ± 0.18 bc	4.20 ± 0.24 a	1.00 ± 0.10 a	20.07 ± 0.40 a	34.64 ± 0.40 a
	1 × 10^6^	-	-	2.00 ± 0.15 a	2.00 ± 0.13 ab	4.38 ± 0.22 a	1.17 ± 0.17 a	20.08 ± 0.56 a	35.00 ± 0.49 a
FCLIL	CK	-	-	-	2.05 ± 0.12 b	3.47 ± 0.21 a	1.05 ± 0.16 a	17.56 ± 0.38 b	31.22 ± 0.56 b
	1 × 10^4^	-	-	-	2.06 ± 0.06 b	3.88 ± 0.15 a	0.75 ± 0.14 a	20.83 ± 0.30 a	34.58 ± 0.36 a
	1 × 10^5^	-	-	-	2.18 ± 0.13 ab	3.93 ± 0.21 a	1.00 ± 0.20 a	21.36 ± 0.41 a	35.55 ± 0.31 a
	1 × 10^6^	-	-	-	2.44 ± 0.16 a	3.85 ± 0.19 a	1.17 ± 0.11 a	21.25 ± 0.48 a	36.25 ± 0.25 a
FCML	CK	-	-	-	-	3.00 ± 0.26 a	1.86 ± 0.38 b	18.67 ± 0.80 b	32.58 ± 0.94 b
	1 × 10^4^	-	-	-	-	2.94 ± 0.32 a	1.71 ± 0.24 b	21.00 ± 0.47 a	34.81 ± 0.52 a
	1 × 10^5^	-	-	-	-	2.81 ± 0.92 a	1.53 ± 0.26 b	22.58 ± 0.61 a	36.08 ± 0.78 a
	1 × 10^6^	-	-	-	-	3.33 ± 0.31 a	3.00 ± 0.53 a	20.71 ± 0.61 a	36.00 ± 0.44 a
FCSML	CK	-	-	-	-	-	2.29 ± 0.38 ab	20.24 ± 0.63 c	33.35 ± 0.71 b
	1 × 10^4^	-	-	-	-	-	2.42 ± 0.29 ab	24.60 ± 0.54 a	37.00 ± 0.49 a
	1 × 10^5^	-	-	-	-	-	1.60 ± 0.31 b	21.85 ± 0.68 bc	38.77 ± 0.61 a
	1 × 10^6^	-	-	-	-	-	2.78 ± 0.32 a	24.00 ± 1.15 ab	36.67 ± 0.88 a
FCPC	CK	-	-	-	-	-	-	19.00 ± 0.91 b	31.43 ± 0.86 b
	1 × 10^4^	-	-	-	-	-	-	22.80 ± 0.85 a	36.67 ± 0.73 a
	1 × 10^5^	-	-	-	-	-	-	24.67 ± 0.80 a	36.58 ± 1.00 a
	1 × 10^6^	-	-	-	-	-	-	23.27 ± 0.55 a	36.27 ± 0.54 a

Treatment: NF: naive adult female wasps, NM: naive *M. alternatus* larvae, FCE: females caring for eggs, FCEIL: females caring for early instar larvae, FCLIL: females caring for late instar larvae, FCML: females caring for mature larvae, FCSML: females caring for spinning mature larvae, and FCPC: females caring for pupal cocoons [22]. POP: pre-oviposition period, ES: eggs of *S. guani*, EIL: early instar larval, LIL: late instar larval, ML: mature larval, SML: spanning mature larval, PC: pupa cocoon, and WG: whole generation. Different letters above the table indicate significant differences (mean ± SE, *n* = 20 in each treatment).

## Data Availability

The original contributions presented in this study are included in the article. Further inquiries can be directed to the corresponding author.
